# Time to Readmission and Its Prognostic Factors among Hospitalised Heart Failure Patients

**DOI:** 10.21315/mjms-01-2025-072

**Published:** 2025-06-30

**Authors:** Yusran Yusoff, Wan Nor Arifin, Sarimah Abdullah

**Affiliations:** 1 CRC Kelantan, Institute for Clinical Research, National Institute of Health, Ministry of Health Malaysia, Selangor, Malaysia; 2 Biostatistics and Research Methodology Unit, School of Medical Sciences, Health Campus, Universiti Sains Malaysia, Kelantan, Malaysia

**Keywords:** heart failure, hospitalisation, patient readmission, prognosis, risk factors

## Abstract

**Background:**

Heart failure (HF) is a common comorbidity in the adult population and a common cause of recurrent readmissions. This study aimed to determine the proportion of readmissions, the median time to readmission and its prognostic factors among hospitalised HF patients.

**Methods:**

This is a retrospective cohort study that involved patients admitted for HF at a tertiary hospital in Kelantan, Malaysia from October 2021 to December 2022. Adult patients who underwent a formal echocardiogram within one year of the index hospitalisation were included. Patients were excluded if they had inpatient mortality or an active malignancy, if they were transferred to another facility, or if they were discharged against medical advice. They had an additional follow-up period of one year to assess the event of interest (readmission). Patients who did not experience a readmission were censored. Prognostic factors for the time to readmission were identified using multiple Cox regression analysis.

**Results:**

A total of 276 patients were included in the analysis, with a mean age of 60.64 years. The proportions of readmissions at six months and one year after discharge were 51.8% and 63.4%, respectively. The median time to readmission for the cohort was 118 days. Prognostic factors for the time to readmission included atrial fibrillation (AF) (adjusted hazard ratio [AHR] = 2.06; 95% confidence interval [CI] : 1.42, 2.99), chronic kidney disease (CKD) (AHR = 1.53; 95% CI: 1.14, 2.04), a low albumin level (AHR = 0.96; 95% CI: 0.94, 0.99), a high aspartate aminotransferase (AST) level (AHR = 1.003; 95% CI: 1.001, 1.006) and ejection fraction (EF) ≤ 40% (AHR = 1.37; 95% CI: 1.03, 1.84).

**Conclusion:**

Most patients experienced a readmission within six months of discharge. Several factors were identified as prognostic factors for readmission. Therefore, clinicians must optimise patient care before discharge, paying special attention to these factors.

## Introduction

Heart failure (HF) is a prevalent non-communicable disease among adults worldwide. It is defined as a clinical syndrome with symptoms and/or signs caused by a structural and/or functional cardiac abnormality and corroborated by elevated natriuretic peptide levels and/or objective evidence of pulmonary or systemic congestion (1). In terms of disease burden, HF can be considered a pandemic, as it affects 64.3 million individuals worldwide (2). Malaysia had one of the highest HF prevalence rates in Southeast Asia, with 721 cases per 100,000 persons in 2017, which is an increase of 7.7% compared to data in 1990 (3). HF has a significant economic impact, which is anticipated to grow as the prevalence of the disease continues to rise. Most of its impact is linked to direct costs, including inpatient care and readmissions. In developed nations, HF accounts for about 1%–2% of all hospital admissions, and approximately 50% of HF patients are readmitted within one year of their initial hospitalisation (2). A cost analysis study in Malaysia reported spending for chronic HF patients as USD 1,971 per patient per year (4).

High readmission rates among HF patients also indicate issues with patient management, discharge planning and follow-up care, which underscores the need for improvement in these areas. Additionally, recurrent hospitalisations disrupt the continuity of care and can hinder the effective long-term management of HF, leading to a vicious cycle of repeated admissions. The instability caused by frequent readmissions exacerbates the patient’s physical health and negatively impacts their quality of life, limiting their ability to perform daily activities and maintain social relationships (5). This diminished quality of life highlights the importance of identifying and mitigating the factors that contribute to repeated hospitalisations. Doing so can improve both the clinical outcomes and overall life satisfaction of HF patients.

Another important issue is the lack of local data on readmission among HF patients. Mohd Ghazi et al. (6) examined readmission among HF patients in a private heart institute, which does not represent the general population. Meanwhile, Lim et al. (7) used a national discharge database that only included demographic characteristics. Another local study by Raja Shariff et al. (8) only reported descriptive data on HF readmission without identifying prognostic factors. This study provides valuable insights that help fill the knowledge gap regarding this issue. The study aims to determine the time to readmission and its prognostic factors among hospitalised HF patients; it considers both demographic and clinical parameters, including laboratory values.

## Methods

### Study Design, Setting and Participants

This was a single-centre retrospective cohort study conducted at a tertiary hospital in Kelantan, Malaysia. The study cohort comprised HF patients admitted during the accrual period from October 2021 to December 2022. The diagnosis of HF was based on the primary diagnosis documented in the discharge summaries, as determined by the treating cardiologists or physicians. The term “index hospitalisation” referred to the first hospitalisation for HF during the accrual period. The event was defined as a readmission during the follow-up period. It was the first acute hospitalisation after discharge from the index hospitalisation. Patients who did not experience readmission were censored; this included those who experienced mortality, were lost to follow-up due to migration or were readmitted to another hospital during the follow-up period. An additional follow-up period of one year (from January 2023 to December 2023) was included to assess the readmission status.

The inclusion criteria were adult patients who had undergone a formal echocardiogram within one year of their index hospitalisation. Patients who experienced inpatient mortality or active malignancy, those who transferred to another facility, and those who were discharged against medical advice during index hospitalisation were excluded.

This study was registered with the National Medical Research Register (NMRR ID-23-02798-FDH [IIR]), and ethical approval was obtained from the Medical Research and Ethics Committee of the Ministry of Health of Malaysia (23-02798-FDH [1]). Ethical approval was also obtained from the Human Research Ethics Committee of Universiti Sains Malaysia (JEPeM-USM). Following ethical approval, permission was obtained from the head of the relevant medical department and the hospital director prior to data collection.

The sample size was calculated using an online sample size calculator based on a single proportion formula for the proportion of readmissions (9). The reported proportion of readmissions at six months after discharge was 24% (10). Precision was set at 6%, and the confidence level was set at 95%. A dropout rate of 10% was factored into the calculation. The minimum required sample size was 217. The sample size for the Cox regression analysis was calculated using the PS software, based on the hazard ratio or relative risk. The type I error and power were set at 5% and 80%, respectively. The median survival of the control group was six months (11). With an expected hazard ratio of 1.45 for chronic kidney disease (CKD), the minimum required sample size was 274 (305 including a 10% dropout rate). No sampling method was employed, as all available cases were selected based on the eligibility criteria.

### Data Collection

A standardised data collection form was used to collect relevant information. Demographic and comorbidity data were collected. Presence of CKD was based on the documentation in the discharge summary and was supported by evidence of renal impairment (an estimated glomerular filtration rate [eGFR] < 30 ml/min based on the 2021 CKD-EPI calculation). Clinical information during the index hospitalisation (e.g., length of stay [LOS], admission to the intensive care unit (ICU), laboratory parameters, vital signs upon discharge and discharge medications) was also gathered.

### Statistical Analysis

Data analysis was performed with R version 4.4.0. Descriptive statistics were used to summarise the socio-demographic profile of the patients. Numbers and percentages were obtained for all categorical variables, while numerical variables were summarised with either means (standard deviations [SDs]) or medians (interquartile ranges [IQRs]), depending on the normality of distribution. The proportions of readmission are presented as percentages with 95% confidence intervals (CI), determined at six months and one year after discharge. The median time to readmission was determined by Kaplan–Meier analysis. Cox regression analysis was utilised to identify prognostic factors for time to readmission following discharge. Variables with a *P*-value below 0.25 based on simple Cox regression analysis were considered for variable selection in the multiple Cox regression analysis. This cut-off value was chosen to allow for a broader inclusion of variables for further analysis and to ensure that potentially important variables were not prematurely excluded (12). Variable selection was performed using a manual method. Subsequently, interaction and multicollinearity between the selected variables were checked. Proportional hazard assumptions were examined using scaled and unscaled Schoenfeld residuals, log-log plots (for categorical variables), and Nelson-Aalen hazard function plots. The final model was presented with adjusted hazard ratios (AHR) and 95% CIs. Statistical significance was set at *P*-value less than 0.05.

## Results

A total of 276 patients were included in the analysis. Most of them were Malays, with a mean age of 61 years (SD = 13). More than half of the patients had underlying hypertension (81%), ischaemic heart disease (62%), type II diabetes mellitus (57.2%) and CKD (50.4%). Hyperlipidaemia (38%) and atrial fibrillation (AF) (17%) were also common. A descriptive summary of the patients’ baseline characteristics is presented in Table 1.

Information regarding the index hospitalisation is summarised in Table 2. The median LOS was four days, and the length was almost identical when comparing the censored and readmitted groups. More than half of the patients (56.9%) had ejection fraction (EF) ≤ 40%. A descriptive summary of medications prescribed at discharge is provided in the Appendix.

**Figure 1:**
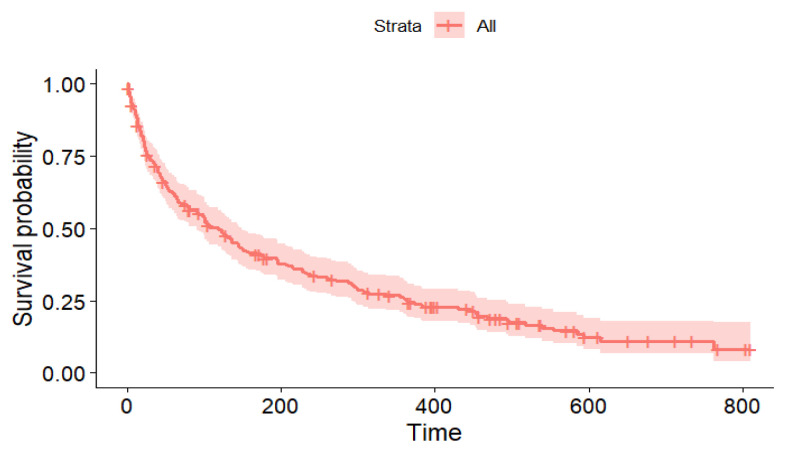
The Kaplan–Meier curve for time to readmission

The total number of readmissions was 193 (70%), with a median time to readmission of 118 days (95% CI: 90, 149). The Kaplan–Meier curve illustrating the time to readmission is displayed in Figure 1. The period from discharge to readmission was categorised into two distinct variables: readmission within six months and within one year after discharge. The proportions of readmission were 51.8% (95% CI: 45.8, 57.8) and 63.4% (95% CI: 57.4, 69.0) at six months and one year following discharge, respectively.

There were twelve variables identified from simple Cox regression analysis with *P* < 0.25 (Table 3). The predictors of time to readmission identified via the multiple Cox regression analysis are presented in Table 4 with AHR and 95% CIs. The final model consisted of five predictors. It fulfilled the proportional hazard assumptions, and no interaction terms were found between the factors. Patients with CKD had a 53% higher hazard of readmission than those without CKD (AHR = 1.53; 95% CI 1.14, 2.04; *P* = 0.004). Patients with AF had more than twice the hazard of readmission compared to those without AF (AHR = 2.06; 95% CI: 1.42, 2.99; *P* < 0.001). Reduced ejection fraction (EF ≤ 40%) was associated with an increased risk of readmission (AHR = 1.37; 95% CI: 1.03, 1.84; *P* = 0.033). A low serum albumin level (AHR = 0.96; 95% CI 0.94, 0.99; *P* = 0.005) and a high aspartate aminotransferase (AST) level (AHR = 1.003; 95% CI: 1.001, 1.006; *P* = 0.022) were also associated with an increased risk of readmission.

## Discussion

In this study, we aimed to determine the time to readmission and its prognostic factors among hospitalised HF patients. We observed a relatively high proportion of readmissions. The proportions of readmissions at six months and one year after discharge were 51.8% and 63.4%, respectively. The median time to readmission was 118 days. The prognostic factors for time to readmission were CKD, EF ≤ 40%, AF, a low albumin level and a high AST level, as identified through the multiple Cox regression analysis.

The proportion of readmissions at six months after discharge (51.8%) was higher than the 24% and 32.9% reported in two studies conducted in Middle Eastern countries (5, 7). A study carried out in Malaysia also reported a lower proportion of readmissions (39.5%) for the same period (8). However, the proportion of readmissions at one year after discharge (63.4%) was lower than that in the Malaysian study, which was 76.1% (8). In two meta-analyses, the proportions of readmissions were 56% and 53% at one year after discharge (13, 14). This suggests that the patients in our study tended to have earlier readmission within six months after discharge. One possible reason for this is the relatively low socioeconomic status of people living in Kelantan (15). HF patients with lower socioeconomic status are considered vulnerable and experience higher rates of hospitalisation and readmission (16).

The median time to readmission was 118 days, which also shows that most of the patients had readmission within six months. Most studies do not report the median time to readmission beyond the typical 30-day readmission window. A study from the China PEACE Heart Failure Study reported a median duration from discharge to readmission of 88 days (17). In another study, the median time to readmission for males was six months, while for females, it was seven months (11). It is important to note that Malaysian HF patients are generally younger, with more severe clinical presentations, and are linked to multiple cardiovascular risk factors (18). They also have relatively short hospital stays, which is typical in heavily burdened Malaysian public hospitals (18). These factors prevent complete symptom resolution and hinder proper patient optimisation and education. This significantly influences the risk and frequency of hospital readmission.

In the present study, we also identified several prognostic factors for time to readmission. Our results show that CKD is an important prognostic factor, which is consistent with the findings of several other studies. Eastwood et al. (19) found that renal disease was a significant risk factor for both 7-day and 30-day all-cause readmissions. A study conducted in the USA reported that abnormal renal function at presentation increased the risk of readmission (20). Furthermore, several researchers have highlighted surrogate markers for renal disease, such as high urea/creatinine levels and low eGFR, as important prognostic factors (6, 10, 21). This finding is not surprising, as patients with renal disease alone are already at high risk of recurrent admissions (22). Concomitant HF complicates the situation as the interplay between the two conditions significantly increases the risk of readmission. This can be explained by the condition known as cardiorenal syndrome.

Another important factor was EF ≤ 40%, also known as HF with reduced EF (HFrEF) according to the HF classification. Habib et al. (21) observed that patients with EF < 30% exhibited a higher risk of readmission. Another study showed that higher EF was associated with a lower risk of readmission (10). However, our result contradicts the finding of Regmi et al. (23), who reported that HF patients with preserved EF had a higher risk of readmission than those with HFrEF. Low EF is known to be related to poor outcomes. Patients with reduced EF tend to experience more severe symptoms, which increases the risk of readmission. In contrast, patients with preserved EF generally have less severe symptoms, better quality of life and lower hospital admission rates (24).

AF contributed to a higher risk of readmission. This finding is consistent with a study conducted in the USA (25). Even among patients with normal EF, coexisting AF is associated with worse clinical outcomes than those of patients without AF (24). These results, coupled with our findings, confirm the significant role of AF in predicting readmission among HF patients. AF is the most common sustained cardiac arrhythmia characterised by irregular heart rhythm. It is prevalent among HF patients and is also linked to other comorbidities, such as CKD, ischaemic heart disease, and diabetes (26). This combination complicates the clinical picture and contributes to a higher risk of readmission. AF can cause adverse haemodynamic instability, which worsens HF and leads to hospitalisation regardless of the baseline heart rate (27). Arrhythmia can also lead to thromboembolic events which are a common cause of readmission (28).

In our study, a low albumin level also increased the risk of readmission. Hypoalbuminaemia is common in HF patients, with two large HF studies reporting prevalence of 18% and 25% (29). The role of albumin levels as a prognostic factor for readmission has not been described in previous studies. However, hypoalbuminaemia has been shown to be associated with poor clinical outcomes. It is linked to higher inpatient and long-term mortality (30). A meta-analysis involving 48 studies also found that hypoalbuminaemia was significantly associated with long-term mortality, particularly one year after discharge (31). Hypoalbuminaemia promotes the progression of HF by increasing the risk of pulmonary congestion as well as worsening myocardial dysfunction and fluid retention (32).

Another prognostic factor identified in the multivariable analysis was the AST level. Elevated AST levels in HF patients are most likely a sign of liver injury. The pathophysiology of liver dysfunction in HF is poorly understood, but the term “cardiohepatic interaction” is also used in relation to cardiorenal syndrome, as described above (33). Hepatic congestion and liver necrosis due to reduced cardiac output are plausible explanations in this context (34). Increased levels of AST are typically associated with hepatocellular damage resulting from ischaemic injury (35). As is the case with albumin levels, AST levels have not been reported as important factors for readmission; however, they are associated with poor clinical outcomes.

Our results do not show that age was an important risk factor for readmission, which contradicts several previous studies. Kaneko et al. (36) and Eastwood et al. (19) found that patients aged ≥ 75 years had a higher risk of readmission, while Aranda et al. (37) reported similar findings for patients aged ≥ 65 years. This could be explained by the relatively younger HF patients in our sample. This aspect is consistent with the findings from the interim analysis of the Malaysian Heart Failure Registry, which showed that HF patients in Malaysia are relatively younger compared to those in Western populations (18). In our cohort, LOS during index hospitalisation was not a predictor of readmission. Previous studies generally categorised LOS and found that patients with LOS > 5 days had a higher chance of readmission (38, 39). Aranda et al. (37) reported a higher risk of readmission among patients with LOS > 7 days. One possible explanation is that the quality and intensity of care during the initial hospital stay, rather than the duration of care, play a more crucial role in determining readmission risk. Moreover, patient-specific factors, such as adherence to treatment, disease severity and comorbid conditions, may have a more pronounced effect on readmission risk than LOS.

The findings of this study have important practical implications for the management of HF patients. The identification of CKD, AF, reduced EF, low serum albumin, and elevated AST levels as significant prognostic factors for earlier readmission can be used to stratify patients according to their risk of being readmitted. It can also guide the development of personalised discharge care and follow-up plans. Patients with these risk factors will benefit from the early involvement of multidisciplinary care teams that include cardiology, nephrology, and nutrition services. Additionally, routine monitoring of laboratory parameters, such as albumin and AST levels, may help identify patients at higher risk who require closer surveillance and proactive intervention. These findings can be incorporated into clinical practice to improve patient outcomes by reducing the risk of preventable readmissions and improving continuity of care after discharge.

This study has several limitations. First, the retrospective design means that data quality relies on the completeness of the clinical records; also, the design does not enable capturing real-time progression of the disease. Second, this was a single-centre study, which means that the proportion of readmissions may have been underestimated due to the inability to trace admissions to other healthcare facilities during the follow-up period. Other factors that may have influenced hospital readmissions were not collected in this study, such as the level of compliance regarding medications and water restriction, as well as important biomarkers (e.g., pro-BNP and uric acid levels). Other important unmeasured confounders were socioeconomic status, health literacy, access to healthcare, and social support systems. Future scholars should aim for wider sampling coverage to ensure adequate representation of the reference population. This is of particular importance, given that HF represents a significant healthcare burden.

## Conclusion

The proportion of readmissions at the study site was higher at six months but similar at one year after discharge compared to the findings of previous studies. The median time to readmission showed a similar pattern. The reasons behind these findings should be understood and discussed with relevant stakeholders to develop strategies for delaying or preventing early readmission. Furthermore, we identified several prognostic factors for time to readmission, including CKD, EF ≤ 40%, AF, low albumin level, and high AST level. These factors should be adequately addressed prior to discharge. Our unique finding regarding the role of albumin and AST levels as prognostic factors for time to readmission warrants further investigation.

## Figures and Tables

**Table 1:** Demographic characteristics of the patients (*N* = 276)

Baseline characteristic	Total *N* = 276 (%)	Censored *n* = 83 (%)	Readmit *n* = 193 (%)
Age (year)^a^	60.64 (13.22)	61.54 (14.40)	60.26 (12.70)

Gender			
Male	148 (53.6)	52 (62.7)	96 (49.7)
Female	128 (46.4)	31 (37.3)	97 (50.3)

Race			
Malay	263 (95.3)	79 (95.2)	184 (95.3)
Non-Malay	13 (4.7)	4 (4.8)	9 (4.7)

Type II DM			
Yes	158 (57.2)	36 (43.4)	122 (63.2)
No	118 (42.8)	47 (56.6)	71 (36.8)

Hypertension			
Yes	225 (81.5)	69 (83.1)	156 (80.8)
No	51 (18.5)	14 (16.9)	37 (19.2)

CKD			
Yes	140 (50.4)	39 (47.0)	101 (51.8)
No	136 (49.6)	44 (53.0)	92 (48.2)

IHD			
Yes	171 (62.0)	52 (62.7)	119 (61.7)
No	105 (38.0)	31 (37.3)	74 (38.3)

Hyperlipidaemia			
Yes	105 (38.0)	24 (28.9)	81 (42.0)
No	171 (62.0)	59 (71.1)	112 (58.0)

AF			
Yes	47 (17.0)	11 (13.3)	36 (18.7)
No	229 (83.0)	72 (86.7)	157 (81.3)

CVA			
Yes	29 (10.5)	9 (10.8)	20 (10.4)
No	247 (89.5)	74 (89.2)	173 (89.6)

Obesity			
Yes	23 (8.3)	5 (6.0)	18 (9.3)
No	253 (91.7)	78 (94.0)	175 (90.7)

a Mean (SD).

CKD = chronic kidney disease; IHD = ischaemic heart disease; AF = atrial fibrillation; CVA = cerebrovascular accident

**Table 2:** Clinical characteristics and laboratory parameters of the patients (*N* = 276)

Clinical characteristic	Total *N* = 276 (%)	Censored *n* = 83 (%)	Readmit *n* = 193 (%)
Length of stay (days)^a^	4.00 (5.00)	5.00 (4.50)	4.00 (5.00)

ICU/HDW admission			
Yes	48.00 (17.40)	19.00 (22.90)	29.00 (15.00)
No	228.00 (82.60)	64.00 (77.10)	164.00 (85.00)

EF (%)^b^	43.21 (16.45)	41.91 (16.75)	43.76 (16.33)
EF > 40	119.00 (43.10)	34.00 (41.00)	108.00 (56.00)
EF ≤ 40	157.00 (56.90)	49.00 (59.00)	85.00 (44.00)

Valvular lesion			
Yes	62.00 (22.50)	18.00 (21.70)	44.00 (22.80)
No	214.00 (77.50)	65.00 (78.30)	149.00 (77.20)

Systolic BP (mmHg)^b^	123.38 (17.74)	123.61 (18.94)	123.25 (17.14)

Diastolic BP (mmHg)^b^	74.98 (12.60)	74.88 (13.96)	75.03 (11.86)

Heart rate (bpm)^b^	79.76 (12.92)	81.27 (11.83)	78.95 (13.44)

CK-MB^a^	19.00 (10.00)	19.00 (15.00)	19.00 (8.00)

Haemoglobin^b^	12.45 (2.40)	12.61 (2.29)	12.37 (2.46)

Albumin^b^	36.64 (5.49)	343.86 (5.34)	34.55 (5.56)

ALT^a^	25.00 (26.25)	32.00 (23.50)	23.00 (25.00)

AST^a^	32.00 (21.00)	33.00 (17.00)	31.00 (22.00)

a Median (IQR);

b Mean (SD).

ICU = Intensive Care Unit; HDW = High Dependency Ward; EF = ejection fraction; CK-MB = Creatine Kinase-MB; ALT = alanine aminotransferase; AST = aspartate aminotransferase

**Table 3:** Simple Cox regression analysis for variables with *P* < 0.25

Variables	β (SE)	Crude HR (95% CI)	Wald statistic	*P*-value
Type II DM				
No	0	1.00		
Yes	0.34 (0.15)	1.41 (1.05, 1.89)	5.32	0.021

CKD				
No	0	1.00		
Yes	0.43 (0.15)	1.51 (1.13, 2.01)	8.52	0.004

AF				
No	0	1.00		
Yes	0.62 (0.19)	1.86 (1.29, 2.69)	11.04	< 0.001

Hyperlipidaemia				
No	0	1.00		
Yes	0.24 (0.15)	1.27 (0.96, 1.70)	2.72	0.099

EF (%)				
> 40	0	1.00		
≤ 40	0.17 (0.15)	1.19 (0.89, 1.58)	1.38	0.241

Urea	0.029 (0.013)	1.029 (1.004, 1.055)	5.31	0.021

Albumin	−0.030 (0.010)	0.97 (0.940, 0.990)	6.6	0.01

ALP	0.005 (0.002)	1.005 (1.002, 1.008)	9.25	0.002

ALT	0.001 (< 0.001)	1.001 (1.000, 1.003)	1.85	0.174

AST	0.003 (0.001)	1.003 (1.000, 1.006)	4.94	0.026

ACEi				
No	0	1.00		
Yes	−0.31 (0.18)	0.73 (0.52, 1.04)	2.99	0.084

Insulin				
No	0	1.00		
Yes	0.28 (0.15)	1.32 (0.98, 1.79)	3.33	0.068

β = regression coefficient; HR = hazard ratio; EF = ejection fraction; ALP = Alkaline phosphatase; ALT = Alanine transaminase; AST = Aspartate transaminase

**Table 4:** Final model for multiple Cox regression analysis

Variables	β (SE)	Adjusted HR (95% CI)	Wald statistic	*P*-value
CKD				
No	0	1.000		
Yes	0.420 (0.150)	1.530 (1.140, 2.040)	2.85	0.004

AF				
No	0	1.000		
Yes	0.720 (0.190)	2.060 (1.420, 2.990)	3.78	< 0.001

EF (%)				
> 40	0	1.000		
≤ 40	0.320 (0.150)	1.370 (1.030, 1.840)	2.13	0.033

Albumin	−0.040 (0.010)	0.960 (0.940, 0.990)	−2.80	0.005

AST	0.003 (0.001)	1.003 (1.001, 1.006)	2.29	0.022

β = regression coefficient; HR = hazard ratio; CKD = chronic kidney disease; AF = atrial fibrillation; EF = ejection fraction; AST = aspartate transferase

## Appendix

**Table:** Medications upon discharge (*N* = 276)

Medications	Total *N* = 276 (%)	Censored *n* = 83 (%)	Readmit *n* = 193 (%)
B-blocker			
No	110 (39.9)	27 (32.5)	83 (43.00)
Yes	166 (60.1)	56 (67.5)	110 (57.00)

ACEi			
No	209 (75.7)	55 (66.3)	154 (79.80)
Yes	67 (24.3)	28 (33.7)	39 (20.20)

ARB			
No	252 (91.3)	78 (94.0)	174 (90.20)
Yes	24 (8.7)	5 (6.0)	19 (9.84)

Loop diuretic			
No	63 (22.8)	22 (26.5)	41 (21.20)
Yes	213 (77.2)	61 (73.5)	152 (78.80)

MRA			
No	179 (64.9)	54 (65.1)	125 (64.80)
Yes	97 (35.1)	29 (34.9)	68 (35.20)

Insulin			
No	193 (69.9)	64 (77.1)	129 (66.80)
Yes	83 (30.1)	19 (22.9)	64 (33.20)

ACEi = angiotensin-converting enzyme inhibitors; ARB = angiotensin II receptor blockers; MRA = mineralocorticoid receptor antagonists
